# ‘Leave Your Ego at the Door’: A Narrative Investigation into Effective Wingsuit Flying

**DOI:** 10.3389/fpsyg.2017.01985

**Published:** 2017-11-17

**Authors:** Cedric Arijs, Stiliani Chroni, Eric Brymer, David Carless

**Affiliations:** ^1^Department of Physical Education & Sports Science, University of Thessaly, Trikala, Greece; ^2^Department of Sports and Physical Education, Faculty of Public Health, Inland Norway University of Applied Sciences, Elverum, Norway; ^3^Institute of Sport, Physical Activity and Leisure, Carnegie School of Sport, Leeds Beckett University, Leeds, United Kingdom

**Keywords:** wingsuit flying, extreme sports, self-knowledge, narrative, elite performance

## Abstract

In recent years there has been a rapid growth in interest in extreme sports. For the most part research has focused on understanding motivations for participation in extreme sports and very little research has attempted to investigate the psychological structure of effective performance. Those few studies that have attempted to explore this issue have tested models designed for traditional sport on adventure sports. However, extreme sports are not the same as adventure sports or traditional sports. This study employed a narrative approach to investigate experiences of effective performance in the extreme sport of proximity wingsuit flying. An overarching theme we labeled ‘leave your ego at the door,’ emerged based on four sub-themes: (1) know thy self, (2) know thy skills, (3) know the environment now, and (4) tame the ‘inner animal.’ These themes are presented and discussed in relation to performance and discovery narratives identified within elite sport, thereby shedding light on how participants’ experiences of the extreme sport of proximity wingsuit flying differ from dominant stories within traditional sports.

## Introduction

Extreme sports are described as sporting activities where a mismanaged mistake or accident would most likely result in death (Brymer and Schweitzer, 2017). While extreme sports share some common ground with traditional sports, that of physical movement, they are different in many ways. In particular, (1) extreme sports involve considerable danger and the potentiality of death (Brymer and Schweitzer, 2013a); (2) extreme sports are not usually competitive in the traditional sense (Breivik, 2010); (3) while examples do exist of extreme sports being undertaken in urban environments (e.g., BASE jumping from buildings) for the most part they take place in the natural environment and demand that the participant engages with the natural environment (Brymer et al., 2010) and, (4) extreme sports are not governed by strict rules, regulations, and constrained performance environments as typically found in more traditional sports (Breivik, 2010). As such, extreme sports present different challenges than many traditional sports, which are compounded by the fact that more broadly adventure sport participation rates seem to be outgrowing many traditional sports (Wheaton, 2004; Pain and Pain, 2005; Brymer and Houge Mackenzie, 2016). Extreme sports are also different from other adventure sports. Though extreme and adventure sports share some larger common ground, that of physical movement taking place in natural environment, they differ in the degree of danger when doing the activity (Brymer et al., 2010; Brymer and Schweitzer, 2017). For example, unlike traditional climbing, extreme climbing does not involve ropes or other protection and support. BASE jumpers jump off solid structures (such as buildings and bridges) without the aid of a second parachute or other safety devices more common in other parachute sports.

The majority of extreme sport research has focused on motivation for participation (Brymer, 2010; Kerr and Mackenzie, 2012) and used traditional theory-driven arguments frequently portraying the participants as thrill-seeking, reckless, self-destructive, and pathological daredevils. This traditional theory driven perspective on extreme sports is most often based on a deficit model and highlights the role of risk and risk taking as the main driver for participation (Brymer, 2010). According to this perspective, extreme sports are solely an outlet for individuals with an innate desire for risky experiences (Farley, 1991; Zuckerman, 2000; Woodman et al., 2010). There are many problems with this approach including that it does not reflect the lived experience of the participants and assumes that the main determinant of effective performance is the innate ability to handle risks (Brymer and Schweitzer, 2017). Recent research on extreme sports has revealed motivations that are more positive and life enhancing and suggests that effective performance is more than the innate ability to handle greater risks (Brymer, 2010; Kerr and Mackenzie, 2012; Brymer and Schweitzer, 2013a,b). Knowledge about these other functions is still limited.

### Performance in Extreme Sports

While research examining effective performance in extreme sports is limited, researchers have started to reflect on how adventure sport athletes (recreational and competitive) perform optimally and in a manner that reduces the likelihood of serious mishap, injury or even death (Kabush and Orlick, 2001; Burke and Orlick, 2003; Griffith et al., 2006; Holland-Smith and Olivier, 2013). For the most part research has focused on applying psychological techniques used in traditional sports to the adventure sports context. For example, studies have investigated the use of imagery in skydiving and rock climbing (Hardy and Callow, 1999; Boyd and Munroe, 2003; Fournier et al., 2008), goal-setting in mountaineering (Bassi and Delle Fave, 2010), mental preparation in skiing (Coleman and Orlick, 2006) and coping skills in a multitude of adventure sports (Young and Knight, 2014). Although these studies provide some valuable insights, the focus has been on investigating the use of psychological skills in adventure sports where death is an unlikely outcome of a mismanaged mistake of accident.

To date no work has been undertaken on extreme sports as defined in the article, or wingsuit proximity flying in particular. It is possible that effective performance in extreme sports is different to adventure and traditional sports and that effective performance in extreme sports does not perfectly match effective performance in traditional sport or adventure. As such, similarly to the development of sport specific models of performance developed in the early days of sport psychology research (Dishman, 1983), it might be import to develop extreme sport specific models of performance. While valuable advances were made testing clinical and educational psychology models in the early years of sport psychology, the special characteristics of sport and relationships within sport required the development of sport specific models to speed progression. That is, findings from studies applying techniques from traditional sports to adventure sports might not reflect the lived experience of extreme sports participants. A decontextualized perspective on psychological skills that is purely about the awareness of and regulation of thoughts, feelings, and behaviors may not be ideal for the extreme sport athlete. Successful extreme sport participation entails more than efficacious task execution, as effective performance is also coupled with avoiding death through interactions with the environment based on a deep understanding of environmental characteristics (Brymer et al., 2010).

### Narrative Research in Sport

Over the past decade, a number of narrative studies have been conducted into the experiences of elite sportspeople (e.g., Douglas and Carless, 2006, 2009; Carless and Douglas, 2009). This research has led to significant theoretical advances offering new understandings around performance, lifestyle, wellbeing and motivation. Because this literature has potential links to the findings and interpretations this study we provide a brief review below.

Across a variety of traditional sports at the elite/professional level (e.g., golf, rugby, swimming, track and field, rowing, hockey) a particular narrative type – the *performance narrative* – has been shown to be dominant (Douglas and Carless, 2015). Stories which follow the performance plot are most common in elite sport culture, and are assumed by many to be the only type of story high-level athletes can legitimately tell. These stories script a particular way of being which revolves around achieving performance outcomes to the extent that performance concerns come to infuse all areas of the athlete’s life. Hallmarks of performance stories include a prioritization of competition, winning or being the best, discipline, sacrifice, hard work, technique, and the relegation of other aspects of life such as relationships, co-operation, enjoyment, exploration, play (see Douglas and Carless, 2009). An alternative narrative type – the *discovery narrative* – has also been identified which prioritizes a different set of values based around exploration and discovery. In this type of story, Douglas and Carless (2015) suggest, the storyteller prioritizes experiences over outcomes, describing a multifaceted self and a life full of people, places and experiences. Play, adventure, fun, feel and a diverse range of experiences characterize these stories. Success is achieved without prioritizing sport over other areas of life and performance outcomes are important only to the extent that they facilitate new experiences, discoveries and explorations (Carless and Douglas, 2009).

### Purpose of the Study

This purpose of this study was to begin to fill the gaps in existing knowledge identified above through a narrative exploration of participants’ experiences of effective performance in the extreme sport of proximity wingsuit flying. Wingsuit flying is a relatively new parachute sport involving a specially designed jumpsuit that facilitates forward motion and directional control. Proximity wingsuit flying has become increasingly popular and is considered the most dangerous parachute sport as it involves flying close to structures at speeds of over 200 mph, which means that the pilot has little time to correct errors (Mei-Dan et al., 2013). Given the increasing popularity of extreme sports, it is important to obtain a clear picture of the psychological processes accompanying effective performance among successful extreme sports athletes. Lessons learnt in this extreme sport context also have the potential to enhance to open up new perspectives on performance related cognition and behaviors in other adventure and traditional sports. The study we present here (part of a larger study on the use and development of psychological practices by proximity wingsuit pilots) focused on understanding the psychological practices experienced extreme sport athletes employ to perform and to reduce the likelihood of serious mishaps. We chose a narrative approach which is premised on the belief that to understand wingsuit pilots’ psychological processes – meanings, motivations, beliefs, and attitudes – it is necessary to take seriously their stories of personal lived experience, and within the lived experience certain practices lie.

## Materials and Methods

A narrative methodology was used to allow the participants to relate, in first-person story form, their experiences, practices and processes over time. A narrative is the general structure, template or scheme people draw upon to tell their personal stories (Smith and Sparkes, 2009). As a research approach in sports it has been used to explore various topics, for instance the coach-athlete relationship, sport identities, and Olympic experiences (e.g., Sparkes and Partington, 2003; Douglas and Carless, 2006, 2015; Jowett, 2008; Carless and Douglas, 2013; Kristiansen, 2013). The proximity wingsuit pilots in this study were asked to share stories from their lives in sport with the specific intention of explicating an understanding of the psychological practices of wingsuit flying.

### Participants

Following ethics approval six pilots (aged from 30 to 45 years of age) from across Europe accepted the invitation to participate. Wingsuit proximity pilots were recruited based on three criteria. The first was that participants were experienced in proximity wingsuit flying, as this would warrant that pilots had a repertoire of relevant experience and events to draw on. The second criterion was participants’ capacity and willingness for reflection. Third, the participants’ English language proficiency needed to be at a level sufficient for understanding the questions asked and verbally communicating personal experiences and opinions with an English-speaking interviewer. Purposeful sampling was used (Patton, 2002). Initially participants were contacted based on demonstrated preparedness to engage in personal reflection, in English, such as discussing their philosophies or the mental aspects of sport (e.g., in previously published interviews, documentaries, or personal videos). Participants were also recruited though snowballing where potential participants were referred to the first author by other participants.

The years of experience as wingsuit pilots ranged from 11 to 22 years. All participants had registered more than 1,000 skydives and BASE jumps and were holders of national and international titles in different parachute sub-disciplines, including wingsuit flying. For confidentiality reasons a more detailed description of each participant is not provided as competitive wingsuit flying consists of a small group of easily identified athletes. The study was approved by the University Ethics Committee of the first author. All participants signed a consent form, before participating in the study, informing them in writing of the study purpose and process as well as their rights and obligations as participants. For anonymity, all participants chose a personal pseudonym to be used in all written reports.

### Data Collection

A semi-structured interview process with open-ended questions was developed and pilot tested to check for content and fluidity. The initial interview questions were developed to guide the interview process by encouraging participants to tell their personal stories about the psychological practices of proximity flying while also allowing for “serendipitous findings” (Strean, 1998, p. 342). The first three authors worked to ensure that the questions were appropriately worded for the audience and the research objectives. The questions were used as a guide rather than as a structured process. The data presented in this paper emerged from responses to questions such as: ‘Please describe in detail how you prepare yourself mentally and physically before a typical jump in wingsuit flying?’ and ‘Please now take me through a typical wingsuit jump as if I were in your mind. What do you think, feel, see, and concentrate on once you get air?’

### Procedure

Pilots were initially identified via the World Wide Web and those who fitted the three inclusion criteria were sent invitations with a summary of the study. While the sport of wingsuit proximity flying is growing it is still anew sport and participants herald from all over the world. For reasons of high mobility and pilot time constraints, interviews were conducted using the communication software Skype. While the use of Skype in research has been critiqued for its limitations in terms of rapport building, its capacity to reach a broad participant group across a wide geographical area in a timely and cost effective manner does open up possibilities that the face to face interview is not able to do. Despite the limitations research has shown that interviews undertaken using Skype are comparable to face to face interviews (Janghorban et al., 2014). In this case, we found that Skype facilitated interviews with expert proximity pilots that we would not have been able to recruit because of geographical, financial and time constraints. With the participants’ permission, all interviews were audio recorded and subsequently transcribed verbatim. Interviews lasted between 45 and 90 min and were conclude when first author and participants considered saturation had met. All interviews began with an outline of the intention of the research project and ended by asking if the participant had anything more they wished to add. Transcripts and additional clarification questions were sent to participants for comments (Culver et al., 2012). Three pilots answered these additional questions in writing, while one pilot agreed to a second interview. The transcribed text of this interview was also sent to the pilot for comments. Two pilots said they did not have the time to respond to the additional questions. All pilots confirmed the final transcripts of their interviews.

### Data Analysis

The initial stage of the data analysis process involved immersion in the data by listening to the interviews and reading the transcripts multiple times before starting the thematic analysis process. Following this, a categorical-content perspective (Hiles and Cermák, 2008) was utilized where the text was first broken down to relatively self-contained areas of content (we isolated segments that related to specific discussions during the interviews) before conducting a thematic analysis. The thematic analysis process followed recommendations outlined by Braun and Clarke (2006) for generating initial codes, searching for themes, reviewing themes, defining and naming themes, and producing the final report. The first author conducted the interviews and undertook the initial stages of the thematic analysis process. During analysis, another experienced qualitative researcher, not involved in the study, provided further regular feedback. Further analysis was undertaken with the other authors to explore the form of the stories participants shared. This allowed the form of individual participant’s stories to be compared and contrasted to the more general performance and discovery narrative types discussed above. The identified themes were then sent to the wingsuit pilots for member reflections (Tracey, 2010). This procedure aimed to enrich the analysis process and ensure that the authors did not have sole ownership or power over the final interpretations (Willig and Stainton-Rogers, 2008).

### Findings

The following sections explicate one theme from a larger study on the extreme sport experience which we have called ‘leave your ego at the door.’ This theme emerged from the analysis of the content of participants’ accounts of their lived experiences of wingsuit flying. Four interrelated sub-themes emerged from the data: (1) know thyself; (2) know thy skills; (3) know the environment now; (4) tame the ‘inner animal.’ Taken together, these themes provide insights into the psychological practices that underpin pilots’ survival (chances of living to fly another day), performance (effective participation in both recreational and competitive flying), and personal rewards (the benefits of wingsuit flying). Below, we consider these themes in turn, referring to pilots by their chosen pseudonym and omitting details that could make them recognizable.

### Know Thyself

Wingsuit pilots stories reflected the ancient Greek aphorism ‘know thyself,’ sometimes described as the greatest form of knowledge, transcending time and context. The aphorism conveys deep knowledge of the self which for Socrates, as described by Xenophon (1923) and Plato (1982) in the Socratic dialogs, was the ultimate form of knowledge guiding a person’s thoughts and actions. All pilots placed emphasis on learning to honestly tune into personal motivations and underlying values in the process of performing optimally. They elaborated on how the development of this kind of awareness is both a proactive and an ongoing process. Awareness of values and motivations is not only needed before undertaking the first wingsuit flight but further supports and guides their development. The ability to ‘know thy self’ underpins and fosters the process of mastering human flight.

For Medusa the continual process of tuning into ‘why’ he participates in wingsuit flying reveals that flying is something fundamental to living, something that he ‘can’t live without’:

I kind of tune into why I’m doing this. Like what is so special about this and why I do this? … I’m doing it because I love what I do and the feeling of flying is something I can’t live without.

The sense of an activity that is experienced as special is also evident in this account from Pinky:

When I’m flying for myself doing something new or just carving through terrain, I like to make noises to myself like [makes flying noises like “whoosh!”]. They enrich my experience as I like go carving past something or flying in between something. Keeps me in the moment and also makes me feel like I’m a child and I’m playing. It’s one of the things adults forget to do is play. So when I’m jumping for myself, I do become a bit of a child, I make noises, I giggle, I laugh, when my parachute opens I scream and yell with joy when I’ve done something that pleases me.

While the *content* of these excerpts offers insights regarding the importance participants attached to knowing themselves, the *form* of the excerpts offers a second level of insight. In this sense, pilots’ stories about ‘knowing thyself’ – as these excerpts illustrate – also deviated (in terms of form) from the dominant performance narrative type. Performance stories revolve around performance outcomes, technique, sacrifice, a future focus, hard work (Douglas and Carless, 2006, 2015). In contrast, Pinky describes how flying “makes me *feel like I’m a child and I’m playing*” while Medusa says “the *feeling* of flying is something I can’t live without.” These examples are not typical performance stories of hard work, discipline, sacrifice and competition. Rather, they follow the contours of the discovery narrative type, which prioritizes experiences of exploration, playfulness, joy, feel, surprise and immersion in the present moment (Douglas and Carless, 2006, 2015).

The discovery orientation evident in pilots’ stories appears to be an important factor also in regard to their ongoing participation. Steve, for example, shared the following story as he reflected on pilots who have died:

It’s always the same question: Is it worth it? Is it giving me enough satisfaction and pleasure to be worth it? The day it would not give me satisfaction and pleasure, I would quit. But it’s hard sometimes, especially this summer with all the accidents. You ask yourself a lot of questions.

His words reveal a practice of continually re-evaluating his connection to flying in order to reflect on meaning and purpose, perhaps as differentiated from something that drives a pilot to push his limits. Meaning and purpose, for Steve, does not revolve around the values of the performance narrative, but instead the discovery values. He explains how the core value that guides participation stems from the question “Is it worth it?” For Steve, the deep internal sense of satisfaction, pleasure, and the meaning he gains from flying are key to participation and if these were not present he ‘would quit.’

An affinity for discovery stories over performance stories was as well evident in participants’ accounts that highlight the ‘processes’ of wingsuiting as being more important than the ‘outcomes.’ Participants recounted how tragic and unnecessary accidents often occur when the pilots are preoccupied with a desire to achieve a spectacular outcome (e.g., fly the most dramatic line before anyone else does and to share the act with the world). Even in competition, survival and life values prevail over pushing boundaries to achieve any short-term victory. Knowing thy self was discussed by all participants as foundational practice that was in danger of being overlooked and skipped due to the rapid pace of the evolution of the sport, its growing popularity, and the ‘get there quickly’ attitude of many potential pilots. This underlined a prediction that in the near future low knowledge and awareness of self might lead to increased casualties. As Christopher said, “Everyone in all extreme sports is skipping the processes now. But with BASE jumping this results in death, not just a broken ankle like in skateboarding.” For Steve, pilots overlook self-knowledge and self-awareness because “lust for life is getting smaller than your lust for attention. It’s an ego problem or it’s just being really unconscious.” Here, participants forewarn of potential dangers from pursuing to the extreme a performance story.

### Know Thy Skills

The theme ‘know thy skills’ reflects participants’ emphasis on the importance of understanding personal capabilities. Valuing technical knowledge is a key characteristic of the performance narrative (Douglas and Carless, 2015) and, in this way, pilots’ accounts sometimes followed the contours of this narrative type. An important distinction, however, is the way that pilots often focused on knowing the *limits* of their technical skills and capabilities, rather than relentlessly seeking to maximize or ‘push’ their skills. This knowledge was seen as critical as making decisions based on a realistic perception of capabilities was described as essential both for performance and for keeping a pilot safe. They suggested flying without this knowledge leads to being out of control. Pinky exemplified this:

You have to know your limits, otherwise you are just out of control. But I do believe that everyone with the right guidance has the capability of flying a wingsuit off a mountain. It’s a very enriching experience, very rewarding; it allows you to focus on the now and live in the moment, forget about your worries in life and I wish that everybody could understand their position in the greater scheme of life.

In this excerpt Pinky describes how learning to evaluate and appreciate personal constraints (both facilitative and restrictive) and working within personal constraints while flying reduces the chances of being ‘out of control.’ His account also communicates a belief that wingsuit flying is less about talent or ability, and more about learning, development and the ‘right guidance.’ Finally, by contrasting flying to everyday life, Pinky related flying to a positive life enhancing experience associated with finding meaning in the ‘greater scheme of life’ which enhances the possibility of leading an authentic life.

All pilots emphasized that it was important to take time to develop capabilities before initiating wingsuit flying. The general attitude toward progression was voiced by Pinky, who emphasized, “slowly stepping it up and realizing that you don’t have to achieve everything in one summer season. You can come back the next season and crunch a little bit harder again.” The importance of ‘slowly stepping it up’ was exemplified by contrasting concerns about what might happen if the journey was ignored in place of the quick fix. As Christopher related:

If you’re pushing your own boundaries too quickly then you’ll be like a horse with blinkers on. People need to go back and do all the steps and remember [it’s] about the journey not the destination, because you can’t get it back once you’ve learned it and learning it is so much fun. Getting your skills up! So people are missing the point. I’d say probably it’s not about having big balls anymore, because the technology is so good. But it’s about arming yourself with knowledge and longevity, and that’s the key to a great career and happiness. It is about learning to see not just what is in front of you but what is all around you, and to not only see it but feel it as well. Sort of like a sixth sense. But you can’t make this happen overnight. The ‘bigger picture’ can take years, if not a lifetime to get sorted.

In this quote, echoes of a discovery narrative are once again evident as Christopher describes the process of learning to wingsuit as a journey rather than an outcome, which leads to an awareness that facilities ‘a sixth sense’ or the ability to ‘feel’ what is happening all around. This is less a performance story of narrow, time-limited, goal-directed focus on a particular outcome or destination; more a discovery story of openness, learning, taking one’s time and prioritizing the journey. For Christopher, it’s “about the journey not the destination.” Christopher’s story transgresses the performance narrative further when he posits that macho attributes are not required and that focusing on performance outcomes can result in reduced awareness of capabilities which ultimately leads to greater danger. Pinky echoed this perspective, stressing that knowing one’s capabilities is an ongoing process because it only takes one mistake to end a pilot’s life: “You can always come back the next day and come a little closer. But you can’t come back the next day if you’ve gone too close.”

So while participants’ stories within this theme share some similarities with performance stories (e.g., valuing personal capabilities and technical knowledge), they also differ in important respects. This difference, we suggest, revolves around the *inclusion* of a broader range of factors that pilots consider to be personal skills. While performance stories tend toward a relatively narrow focus on the self and one’s technical capabilities (see Douglas and Carless, 2015), pilots’ discovery stories portray a broadening, outward-looking way of being. Moving toward a discovery plot, the stories collected from these wingsuit pilots extend conceptions of what constitutes ‘skill’ in elite performance beyond the technical capabilities that a person possesses (as articulated in performance stories), toward deeper, more profound understanding of personal abilities. Pilots’ conceptions of ‘skills’ thereby incorporates values, processes, insights and personal motivations align more closely with discovery stories.

### Know the Environment Now

A strong thread in participants’ accounts was the need to know – to tune into and be aware of – the environment in which they find themselves at a particular moment in time. Participants described how attending to the external environment was essential for successful flying and that this awareness was actively developed rather than an innate or inbuilt ability. The pilots were adamant that ‘tuning into’ the present moment was not solely about connecting with internal states but also about being ‘connected with the environment.’ Steve exemplified the importance of this awareness:

In a sport like ours it’s a question of survival. If you’re not perfectly in the present moment, if you’re not ultra-connected with your environment, like an animal who’s hunting, you take many more risks. Being able to be perfectly in this present moment will make you much more aware and basically much more efficient in what you are doing.

Steve vividly expressed this intense state of tuning into momentary environmental fluctuations with the metaphor ‘like an animal who’s hunting.’ He is acquainted with the reality that his sport can result in death, and because of this, survival heavily depends on being attuned to potentially significant environmental factors. Dakota described how attending to the environment requires ‘noticing’ – focusing on and attending to – a broad range of environmental factors:

I’m usually assessing the conditions so I’m noticing everything around me, if there’s clouds, I’m noticing the trees, and if there’s wind blowing, noticing if the birds are flying, where they’re flying, how they’re flying because that can give me a good idea if there are any thermals in the air or a decent breeze. Because all of this is going to affect me once I actually jump.

The performance narrative, according to Douglas and Carless (2015), is a story about overcoming through power and/or superiority, of striving to win regardless of the obstacles you may encounter – commonly by considering that obstacles are there for all and it is about the survival of the fittest (mentally and physically). In a performance story, they suggest, the athlete may be understood to be *in opposition* to all obstacles to success (competitors, conditions, injury, etc.). Yet these pilots’ stories reveal a sense of trying to *connect* with – co-operate with – potential obstacles (such as weather conditions).

Steve described how his experience of time changes through the need to notice and weigh this ever-changing range of environmental factors:

You’re like in a tunnel. This 1 min seems to be 5 min. There’s distortion of time, you are super sharp in your senses. In fact to me it’s optimal focus with letting go of everything. Because you have to be in the present moment. There is no way you can multitask in such a survival-based situation.

The outcome of this process of noticing and weighing environmental factors prior to a flight is sometimes the decision to not jump at all. Bumblebee recounted how an experienced wingsuit pilot’s decision *not* to jump had a powerful influence on his own decision making:

A lot of times you might hike hours and hours to get somewhere, blood sweat and tears to get to an exit point and then the weather conditions just aren’t right. Now a lot of people would [jump] anyways, whereas he was the guy that had no problems turning around and then hiking back down. And I remember the first time that happened in [location]. I was just shocked. I was just like, “But we’re up here, it’s not that bad…”, and he was just, “I’m not feeling it (…) the winds are this and that, it’s just not a good idea to go so I’ll hike down and see you at the bottom.” Just like “Waw!” You know, somebody like that I really looked up to in the sport of proxy flying had that kind of willpower. It really stayed with me.

Here again, the narrative form of participants’ stories under this theme also contrast with the plot of performance stories. Pilots stories do not revolve around ‘conquering’ or ‘overcoming’ the environment or the weather conditions (as a performance story likely would), but instead they describe becoming aware of and responsive to these variables. Pilots’ stories portray a sensitivity and responsiveness to what is going on around them. While the performance narrative might be understood to require performance *independent of* or *despite* external factors, pilots’ stories follow a different kind of narrative plot which prioritizes awareness, responsiveness and co-operation.

### Tame the ‘Inner Animal’

While awareness and preparation during the time before the jump is important, a successful jump also relies on a dynamic process of continual re-assessment while flying. The theme ‘tame the inner animal’ reflects an ongoing awareness of one’s inner states as well as environmental boundaries and individual capabilities that combine to support effective decision-making while actually flying. For instance, Bumblebee explained that, for him, success (i.e., effective performance) in wingsuit flying depends on the ability to manage the desire to push beyond one’s capabilities:

You can tell yourself everything you want when you’re on the cliff edge, but once you jump and you start flying most people have that animal taking over where you [tell yourself] “You can make that turn, you can make that corner, you can make this” and you just “Go, go, go, charge!” And the people that live through this world are the ones who in that moment of nanoseconds and the surreal experience, have the mental power to realize that their inner animal is trying to convince them to maybe push it too far. So for me, being on a cliff edge, fighting that emotional battle of [thinking of] the whole family [back home] and “What am I doing?” is the glue that keeps me aware of that animal inside. That helps me not be that guy that tries to drag my toes through the trees, but instead stay ten feet off the trees.

Here Bumblebee used an animal metaphor to articulate the intense conflict that arises during flight. He described how a successful pilot is aware of two contradictory elements that need to be actively managed in order to constrain the craving to push beyond limits and potentially tempt death. According to Bumblebee a pilot’s awareness and mental strength must work collectively within ‘nanoseconds’ to preserve safety. Christopher also articulated this conflict and emphasized that being aware of inner states and personal capabilities enhanced safety for him:

I try not to push past my limits, I try and just run it 50–70% all the time to ensure my safety. I don’t run it a 100%. I could do so much more with my abilities and just my skills over the years, but I’ve seen so many of my friends die because they were running at 100% that I don’t want to do that. I just walk away. If you’re running at a 100% then you’ve got nothing left. And then if you hit a flat spot on a wingsuit flight and you’re running at a 100%, you are in big trouble. I think you’ve got to leave your ego at the door with a lot of these sports.

### ‘Leave Your Ego at the Door’

In the preceding excerpt Christopher used the phrase ‘*leave your ego at the door’* to express concisely and evocatively a notion that other participants also raised, that a successful flight is about holding back, to perform optimally *within* maximum capacity. This act of balance between awareness and regulation means that while the pilot knows he can do more he deliberately retains a margin which provides the capacity to resolve unexpected issues. All interviewed pilots reported that it was imperative to be aware of skills and to accept limits at all moments during the flight. We see this point as important and, once again, something of a deviation from culturally preferred and endorsed performance stories in sport such as ‘no pain, no gain’ or ‘just do it.’ These wingsuit pilots stories are about *not* ‘pushing to the max,’ *not* ‘just doing it’ in conventional elite traditional sports terms, to instead be aware and judicious of the four themes discussed above – in essence: oneself, one’s skills, one’s immediate environment, and one’s ego.

In this sense, ‘leave your ego at the door’ may be understood as a *foundational way of being* for wingsuit pilots which starts (and progresses) with the deliberate intention to tune into personal motivations and values as well as skills and capabilities. To achieve this foundational way of being, pilots’ accounts suggest that the four sub-themes discussed above needed to realized. In other words, when pilots were able to attend to knowing themselves, knowing their skills, knowing the environment and taming the inner animal, they were able to ‘leave their ego at the door’ and perform optimally. This way of being has a temporal configuration characterized by an active process of becoming and being increasingly aware, sensitive, conscious, attentive, alert, and responsive to self, skills, environment and motivations. While the essence of the experience happens during each flight, the process required for effective flying can take years to develop. This development is active and ongoing, perhaps most clearly evident when pilots described their personal preparations for flight. As Bumblebee exemplified, the preparation process includes reflections on personal values and meanings – an ongoing connection with his being – that keep his flying safe. He shared how the conflicting emotions he feels before jumping, tempered by thoughts of his family, mediate what he does while jumping:

For me, being on a cliff edge, fighting the emotional battle of [thinking about] the whole family [back home] and “What am I doing?” is the glue that keeps me aware of that animal inside me. This helps me not to be that guy who tries to drag his toes through the trees, but instead stays ten feet off the trees.

Medusa also described how he deliberately attempts to center himself and to be at one with the self and the environment:

I like to actually go and close my eyes at the exit point that I’m going to jump [off] and take deep breaths and feel the air, which way the air is moving. Feel the air toward my body. It helps me calm down and it helps me tune my mind and instead of being overamping I’m calmer.

Medusa deliberately evokes a state of calmness before he jumps via a routine that generates both an internal and environmental focus. All pilots reiterated that the process of seamlessly connecting with both the self and the environment is developed over time, as the following accounts illustrate:

I center my body and mind. I think it is something that takes time to learn. You need to be at one with yourself and [to] put all your energy into the one moment of leaping off the object, this way you are in tune 100% with your surroundings and yourself and the present moment. (Christopher)

The most important thing for me personally is to not jump until I’ve calmed all my nerves. And [I] found a way to get my heartbeat to slow down to normal, by breathing deep, have that kind of feeling of Zen and not be rushed into a moment of jumping before you’re ready. That’s probably the most important part, [to] have your mind in the right place before you exit. (…) I go from a point of nervous, where it feels like a pinball in my head, it’s bouncing around and there’s a lot of nerves and I’m excited and I feel the adrenaline, to eventually through breathing I get to a point of just a calm. (…) It’s not nervousness and excited, you’ve finally found a way to change that into what almost feels happy and warm. Like a place of peace. (Bumblebee)

In summary, all pilots identified three instrumental aspects of present-moment awareness. First, being fully in the present was vital for execution. Second, present-moment awareness is simultaneously an internal focus and an attunement with the environment. Third, present-moment awareness is deliberately cultivated as a key aspect of flying optimally. This kind of ‘pre-flight practice,’ as described by participants above, does not seem to be a replication of the traditional pre-performance routines (Cotterill, 2010, 2011; Cotterill et al., 2010; Yancey et al., 2011) and arousal reduction techniques (Thelwell et al., 2006; Guillot and Collet, 2008; Gucciardi et al., 2010) encouraged in conventional sporting practice. While this pre-flight practice brings the pilots to the desired mental and physical state for optimal execution, it appears to be a notably different kind of experience, at times drawing elements of the dominant performance narrative, but more often following the contours of the less frequently heard (in sport) discovery narrative.

## Discussion

This paper aimed to elaborate on the psychological practices proximity wingsuit pilots employ to perform effectively and to reduce the likelihood of serious mishaps through taking seriously pilots’ stories of their lived experience. A surprising finding is that the form of pilots’ stories deviate in fundamental ways from the dominant performance narrative in sport where the overriding focus is on achieving performance outcomes (see Douglas and Carless, 2006, 2015). Instead, these pilots’ stories follow more closely the contours of the discovery narrative type (see Douglas and Carless, 2006, 2015). This distinction is significant as the wingsuit pilots in this study are achieving high-level or elite performance *without* adopting or subscribing to the values of the culturally dominant narrative type in elite sport. By exhibiting instead the plot of the discovery narrative, their stories map another way to *be effective* at the elite level. This finding contributes to and extends existing narrative research in elite sport and suggests that further research into the experiences of extreme sport participants has the potential to contribute valuable new insights to the field of sport psychology.

Perhaps central to the performance/discovery distinction we have identified is how, for these elite wingsuit pilots, success (such as executing effective jumps) is less about achieving performance outcomes (such as winning or being the best), and more about living to fly another day. As one participant put it, “In a sport like ours it’s a question of survival.” Jenkins (2013) notes the prevalence of battle or war metaphors in sports stories which, according to Douglas and Carless (2015), serve to raise the stakes of sporting contests, portraying winning as a matter of life or death. They observe how, “in war stories, winning is survival while losing is death,” while “in sport whether or not a team or individual wins or loses is *not* a matter of life or death” (Douglas and Carless, 2015, p. 13). The perspective for these wingsuit pilots, however, is that a poor outcome can and does result in death. We propose that this core existential contrast (between sports in which participants do not typically risk death and winsguit flying where they do) *calls for*, *supports* and perhaps even *requires* participants to narrate their lives in ways that are at odds with the dominant narrative within sport.

According to the wingsuit pilots in this study, on any given day the active stage of flying is about knowing, accepting and regulating the relationships between individual features, task features, and environmental features. This balanced co-existence of awareness, acceptance, and regulation led us to choose the expression *leave your ego at the door* to encapsulate a way of being and doing that these participants narrated and endorsed. Critical to this phrase, are the four themes of knowing self, skills, environment and taming one’s ‘inner animal.’ On the basis of participants’ accounts, a wingsuit pilot would not be able to ‘leave his ego at the cliff’s door’ if all these other types and levels of awareness were absent.

In contrast to current research on self-awareness in extreme sports which suggest that extreme sport athletes deliberately avoid any attempt to be self-aware (Castanier et al., 2010) proximity pilots in this study went to great lengths to stress the importance of self-knowledge. Self-awareness is viewed as an essential element of successful flying. To an extent, the pilots in this study described psychological elements that mirror Vealey’s (2007) model of mental skills which emerged based on traditional sports, in that they emphasized the importance of developing self-awareness over time, tuning into the present moment just before the activity, and the continual process of attunement while flying. However, the pilots in this study emphasized the importance of a dynamic person-environment interaction and value-guided action (to walk away and try again at another time) over any effort to avoid or attempt to change the uncomfortable present-moment awareness associated with not being ready for a particular jump (either because of present moment conditions or their skill level). This continuous dynamic person-environment interaction and value-guided action takes the wingsuit performers many steps away from the traditional elite sport unconditional competition for the survival of the fittest. Further, they emphasized the importance of performing well within personal capabilities in order to avoid tempting death.

In the introduction we suggested that understanding the experience of effective performance in extreme sports could help inform extreme sport athletes and perhaps even add to traditional sports. Findings from this study suggest that effective extreme sports participation is not about innate abilities, who can take the biggest risk or who can demonstrate the most macho line. Athletes interested in extreme sports ought to spend time cultivating self-knowledge and guiding values before taking part. Rather than pushing the limits, effective participation is dependent on honest continual self-evaluation and the courage to walk away and participate well within personal capabilities. While majority of traditional sports do not usually involve experiences that might end in death there are still some learnings that might be of use. Considering that the interviewed pilots’ pre-flight practice brings them to an optimal mental and physical state (e.g., focused, absence of heightened pre-execution anxiety) that leads to effective jump execution, traditional performing might be also effective if guided by deep values and close connection with ‘thy self.’ Moreover and in view of recent death cases post athletic retirement, traditional athletes’ lives (during and after sports) might be more prosperous if winning and traditional way of being and doing for elite achievement were put into a different perspective (not the ultimate and only goal), one cultivated by meaning and purpose.

## Conclusion

Self-knowledge, the ability to tune into the environment and the courage to walk away are clearly important elements for the safe and optimal execution of proximity flying. Pilots in this study highlighted the importance of values, of a proactive process of tuning into the self and environment before flying and of an ongoing process during flight, the ultimate aim being to live to fly another day. Findings from this study suggest that far from avoiding self-awareness, self-awareness is vital for effective participation. Successful proximity flying does not seem to depend on an innate ability but the cultivation of meaning and purpose supported by values that reach beyond the activity and stretch into life. Effective participation – and long-term one – is dependent on the development of profound knowledge of the relationship between self, task and the environment. In these ways, we suggest that wingsuit pilots’ stories of their experiences differ markedly from the dominant performance narrative in sport. Instead, the more closely resemble the discovery narrative type, scripting an alternative way of storying, navigating and living life as an elite performer.

## Ethics Statement

This study was carried out in accordance with the recommendations of University of Thessaly’s Ethics Committee with written informed consent from all subjects. All subjects gave written informed consent in accordance with the Declaration of Helsinki. The protocol was approved by the ‘name of committee.’

## Author Contributions

CA lead the project, gathered the data and was instrumental in developing the article, the topic area and interpreting the data. EB was instrumental in developing the article, the topic area and interpreting the data. SC was instrumental in developing the article, and interpreting the data. DC is a narrative specialist and was instrumental in interpreting the data from a narrative perspective.

## Conflict of Interest Statement

The authors declare that the research was conducted in the absence of any commercial or financial relationships that could be construed as a potential conflict of interest.
